# Disrupted hypothalamic functional connectivity related to cognitive impairment after diffuse axonal injury: Retraction

**DOI:** 10.1097/MD.0000000000030346

**Published:** 2022-08-12

The article “Disrupted hypothalamic functional connectivity related to cognitive impairment after diffuse axonal injury”,^[1]^ which published in Volume 100, Issue 48 of *Medicine*, is being retracted due to academic misconduct. After an investigation into reader concerns, the Editorial Office found that passages had been directly plagiarized and/or slightly modified from “Functional plasticity in lateral hypothalamus and its prediction of cognitive impairment in patients with diffuse axonal injury: evidence from a resting-state functional connectivity study”.^[2]^
